# Correlation between Metabolic Syndrome and Intracranial versus Extracranial Arteriosclerosis among Chinese Patients with Stroke

**Published:** 2019-11

**Authors:** Chunyu LIU, Xiaodan YANG, Changqing CHEN

**Affiliations:** Department of Neurology, PLA 254 Hospital, Tianjin 300141, P.R. China

**Keywords:** Metabolic syndrome, Intracranial arteriosclerosis, Extracranial arteriosclerosis

## Abstract

**Background:**

We aimed to explore the correlation between metabolic syndrome and intracranial and extra-cranial arteriosclerosis. Overall, 318 over 60-yr-old patients with cerebral infarction or TIA who were examined by digital subtraction angiography (DSA) in our hospital were enrolled in the study.

**Methods:**

Overall, 192 patients with intracranial and extracranial arteriosclerosis were admitted to the case group (the intracranial and extracranial arteriosclerosis group). Also, 196 patients, suffering from the same condition, were selected from our outpatient clinic and enrolled in the control group.

**Results:**

The prevalence of metabolic syndrome was 31.4%. The prevalence of each metabolic syndrome component in the intracranial arteriosclerosis group was higher than those of the extracranial arteriosclerosis and the control groups. The average component values in the intracranial arteriosclerosis group was higher than those observed in other groups. The prevalence rate of metabolic syndrome had no significant difference among different degrees of stenosis for extracranial arteriosclerosis group. There was a remarkable correlation between intracranial arteriosclerosis and metabolic syndrome (*P*<0.001), while no correlation was detected between extracranial arteriosclerosis and metabolic syndrome (*P*<0.001). We concluded that metabolic syndrome may increase the prevalence risk of intracranial arteriosclerosis. There was a significant correlation between intracranial arteriosclerosis and metabolic syndrome components including hyperglycemia and hypertension. Also, there was a significant correlation between extracranial arteriosclerosis and metabolic syndrome components including hyperglycemia.

**Conclusion:**

We believe that at least three components of metabolic syndrome can obviously increase the risk of intracranial arteriosclerosis.

## Introduction

Acute cerebrovascular disease is one of the important causes of mortality and disability all over the world. There are 7 million known cases of acute cerebrovascular in China, with annul prevalence rate of 260-719 for 10 million people. Every year, 1.65 million patients die from acute cerebrovascular disease (1). The disability caused by cerebrovascular disease is around 75%. At present, the incidence rate of acute cerebrovascular disease is increasing by 8.7% every year. Acute cerebrovascular disease featuring high incidence rate, death rate and disability rate brings a burden to society and families. 87% of patients with acute cerebrovascular disease suffer from ischemic stroke (2). Intracranial and extracranial arteriosclerosis (AS) is an important pathological basis of ischemic stroke (3). Back in 1990s, several studies were conducted on the distribution of intracranial and extracranial arteriosclerosis for different races, and results showed that the distribution of intracranial and extracranial arteriosclerosis had a racial difference. Asian arteriosclerosis lesion always happens to intracranial artery, which is the main reason of ischemic stroke, while the cases in west are just the opposite (4–8). Due to the difference in risk factors and mechanism that can induce intracranial and extracranial arteriosclerosis (9–11), similar treatment methods may have dissimilar effects on the changes to intracranial and extracranial arteriosclerosis.

Metabolic syndrome describes a phenomenon that multiple pathogenic factors aggregate in an individual. As a group of metabolic disorder syndrome, it consists of obesity, hypertension, pathoglycemia and dyslipidemia. In recent years, with the aging population in China as well as the lifestyle of surfeit and sedentariness, the prevalence rate of metabolic syndrome is gradually increasing. The results of the latest nationwide metabolic syndrome research baseline (Inter Asia, 2010–2012) showed that the prevalence rate of metabolic syndrome is 11.0 % (12). All components of metabolic syndrome can increase the risk of arteriosclerosis, at the same time, the risk of arteriosclerosis occurrence is higher when many abnormities combine. There is a difference between intracranial and extracranial arteriosclerosis and metabolic syndrome, as well as the correlation among difference components (13, 14). However, the relevant reports on this difference for Chinese population are very rare.

We aimed to discuss the correlation between intracranial and extracranial arteriosclerosis and metabolic syndrome as well as its relevant components respectively. We conducted cross-sectional study on the patients with intracranial and extracranial arteriosclerosis, in order to provide objective basis for prevention and treatment of ischemic cerebrovascular disease in future.

## Materials and Methods

### Source of Crowd

From September. 2015 to September 2017, 318 over 60-yr-old patients with cerebral infarction or TIA were examined by digital subtraction angiography (DSA) in our hospital. According to the inspection result of DSA that distinguished intracranial arteriosclerosis and extracranial arteriosclerosis, 192 patients with intracranial and extra-cranial arteriosclerosis were enrolled in this study as the case group (intracranial and extracranial arteriosclerosis group). This included 105 patients with intracranial arteriosclerosis and 87 patients with extracranial arteriosclerosis. Overall, 196 patients, suffering from the same condition, were selected from our outpatient clinic and enrolled in the control group. Clinical general information and blood vessel image data were collected from all patients. Blood samples were used for metabolic syndrome predication.

### Criterion of Intracranial and Extracranial Arteriosclerosis

The stenosis of intracranial and extracranial arterial lumen ≥30% was deemed to be arteriosclerosis lesion. The calculation of arterial stenosis rate referred to the measurement standard of North American Symptomatic Carotid Endarterectomy Trial (NASCET) (15): arterial stenosis rate= [ 1 - (inside diameter of blood vessel at the most narrow / inside diameter of normal blood vessel at the far-end stenosis] ×100%.

### Exclusion Criteria

1. Cerebrovascular malformation, aortic dissection, vasculitis;
2. Potential cardiac embolism;
3. Cerebral infarction with unknown cause;
4. Degree of artery stenosis less than 30%, combined with intracranial and extracranial arteriosclerosis;
5. Tumor, infection and hypercoagulation;
6. Severe visual and auditory dysfunction and aphasia (RVR<6 points)
7. Incomplete medical history;
8. Refusal for informed consent signature.

### Diagnostic Criteria

1. Diagnostic Criteria of Metabolic Syndrome IDF standard (16) was applied to metabolic syndrome diagnosis which met these 3 items: 1) Central obesity: the waistline of male ≥90cm, the waist-line of female ≥80cm; 2) Triglyceride (TG) level increasement: > 1.7 mmol/L or acceptance of special treatment on the lipid abnormity; 3) High density lipoprotein cholesterol (HDL-C) level reduction: male < 1.03 mmol/L, female < 1. 29 mmol/L or acceptance of special treatment on the lipid abnormity; 4) Elevated blood pressure: systolic pressure ≥130 mmHg or diastolic pressure ≥85 mmHg, or acceptance of treatment due to the previous diagnosis as high blood pressure; 5) Fasting blood-glucose increasement: Fasting blood-glucose≥100 mg/dl (5. 6 mmol/L) or diagnosis as type 2 diabetes mellitus.

2. Hypertension: with a chronic hypertension history, systolic pressure ≥140 mmHg, diastolic pressure ≥90 mmHg; or only systolic pressure ≥140 mmHg after admission to hospital.

3. Glycuresis: with a diabetes history, fasting blood-glucose ≥7.0 mmol/L, random blood glucose ≥11.l mmol/L after admission to hospital.

4. History of Coronary Heart Disease: with an atrial fibrillation and ischemic heart disease history, or prompt of atrial fibrillation and myocardial ischemia by electrocardiography after admission to hospital to make a definite diagnosis.

5. Smoking: smoke ≥10 cigarettes, for ≥5 years, and still smoke now.

6. Hyperhomocysteinemia: homocysteine ≥15umol/l.

### Main Examination Methods

1. Measurement of Abdominal Girth and Blood Pressure:

When patients stood still and kept eupnea, the diameter from 1 cm above navel was measured horizontally; then patients took a sitting position twice for blood pressure measurements by cuff type mercurial sphygmomanometer.

2. Blood Extraction and Laboratory Examination Methods:

Two samples of 5 ml fast venous blood were extracted from all patients within 24 hours after admission to hospital, and two samples of 5 ml fast venous blood were extracted during medical examination. Oxidase method was used to measure TG/CHO; for the measurement of HDL, precipitator was applied for precipitation and cholesterol levels were measured in supernatant using enzymic method. LDL levels were measured using Friedwald formula (LDL=CHOHDL-TG/5) and glucose oxidase method was used to measure fasting blood glucose.

3. DSA Examination: AdvantxLCA+/LCV+/LC+ angiography machine from America GE company was used and voltage was set as 80 KV, magnitude of current was 500mA and F1 was 75 KV. Seldinger method was adopted to femoral artery puncture tube, with contrast medium of iohexo 300 mgI/ml, to inject by high pressure injector. The condition of common carotid artery arteriography was: flow velocity 5 ml/s, flow volume 7 ml and pressure at 150 Psi.

### Statistical Process

SPSS 18.0 (Chicago, IL, USA) was applied for statistical analysis. Baseline population statistics and the continuous variable of laboratory data was expressed by Mean±standard deviation, and absolute variable was showed by frequency. The comparison of continuous variable among groups was detected by *t* or analyzed by variance, while the comparison of absolute variable was tested by Chi-square. Multi-factor Logistic analysis of regression was used for the research on correlation between intracranial and extracranial arteriosclerosis and metabolic syndrome as well as its components. The results were signified by OR value and 95% confidential intervals. *P* < 0.05 meant a statistical significance.

## Results

### Basic Information of Research Population

Among 388 patients, there were 250 males and 138 females with average age of 65.8±9.3 years old. The comparison of the number the male patients between the intracranial and extracranial arteriosclerosis and the control groups showed no significant difference. There was also no significant difference between the intracranial arteriosclerosis group and the extracranial arteriosclerosis group in terms of the number the male patients. In the intracranial and extracranial arteriosclerosis group, age, smoking, BMI, hypertension, diabetes and prevalence rate of metabolic syndrome were obviously higher than those of the control group (*P* < 0.05, *P* < 0.01). There was no significant difference in age, smoking pattern, BMI, hypertension, diabetes and prevalence rate of metabolic syndrome between the intracranial arteriosclerosis group and the extracranial arteriosclerosis group. The prevalence rate of metabolic syndrome was 31.4%. The prevalence rate of each metabolic syndrome component in the intracranial arteriosclerosis group was higher than those of other groups. The average component number of metabolic syndrome in the intracranial arteriosclerosis group was higher than those of the extracranial arteriosclerosis and the control groups (Table 1).

**Table 1:** Baseline Features of Research Population with Intracranial and Extracranial Arteriosclerosis

***Variable***	***Control Group n=196 (%)***	***Intracranial and Extracranial Arteriosclerosis n=192 (%)***	***Intracranial Arteriosclerosis n=105 (%)***	***Extracranial Arteriosclerosis n=87 (%)***	**P *Value***
Age (yr)	62.67 ± 10.6	67.9±9.5	67.7 ±8.8	68.2 ± 10.4	0.009
Gender, Male	119 (60.7)	131(68.2)	73 (69.5)	58 (66.7)	0.376
Smoking	41(20.9)	65(33.9)	33 (31.6)	32 (36.8)	0.012
Coronary Heart Disease	4 (2.0)	18 (9.4)	5 (4.8)	13 (14.9)	< 0.001
Hypertension	17 (8.7)	117(60.9)	70 (66.7)	47 (54.0)	< 0.001
Diabetes	13 (6.6)	97(50.5)	57 (54.3)	40 (46.0)	< 0.001
Metabolic Syndrome	28 (14.3)	94(49.0)	56 (53.3)	38 (43.7)	< 0.001
Components of Metabolic Syndrome					
Hypertension	23 (11.7)	136(70.8)	80 (76.2)	56 (64.4)	< 0.001
Hyperglycemia	48 (24.5)	117(60.9)	68 (64.8)	49 (56.3)	< 0.001
Hypertriglyceridemia	50 (25.5)	74(38.5)	45 (42.9)	29 (33.3)	0.008
Low High-density Lipoprotein	35 (17.9)	97(50.5)	59 (56.2)	38 (43.7)	< 0.001
Abdominal Obesity	75 (38.3)	119(62.0)	75 (71.4)	44 (50.6)	< 0.001
Metabolic Syndrome (Numberof Component)	1.66 ± 1.32	2.31±1.56	2.55 ± 1.44	2.11 ± 1.52	< 0.001
1	59 (30.1)	33(170.2)	14 (13.3)	19 (21.8)	
2	50 (25.5)	38(19.8)	24 (22.9)	14 (16.1)	
3	25 (12.8)	48(25.0)	28 (26.7)	20 (23.0)	
4	16 (8.2)	31(16.1)	18 (17.1)	13 (14.9)	
5	6 (3.1)	15(7.8)	10 (9.5)	5 (5.7)	

### Different Metabolic Syndrome Distribution of Different Degrees of Intracranial and Extracranial Arteriosclerosis Stenosis

In intracranial Arteriosclerosis group, there were 26 patients (52.6%) with metabolic syndrome among patients with degrees of stenosis ≥70%. There were 30 patients (44.8%) with metabolic syndrome among patients with degrees of stenosis between 30% and 69%. The prevalence rate of metabolic syndrome had a significant difference among different degrees of stenosis (*P*< 0.05). Number of patients with degrees of stenosis ≥70% suffered from hypertension, hyperglycemia, hypertriglyceridemia, low high-density lipoprotein and abdominal obesity were 34, 30, 19, 23 and 28 respectively. Number of patients with degrees of stenosis between 30% and 69% suffered from hypertension, hyperglycemia, hyper-triglyceridemia, low high-density lipoprotein and abdominal obesity were 46, 38, 26, 36 and 47 respectively. Finally, number of patients with degrees of stenosis ≥70% suffered from hypertension and hyperglycemia was obviously more than that of patients with degrees of stenosis between 30% and 69%, and the number of patients with degrees of stenosis ≥70% suffered from 3, 4 and 5 components of metabolic syndrome were apparently higher than those of patients with degrees of stenosis between 30% and 69% (*P*<0.05).

Extracranial Arteriosclerosis Group: The prevalence rate of metabolic syndrome had no significant difference among different degrees of stenosis and the number of patients with degrees of stenosis ≥70% suffered from hypertension, hyperglycemia, hypertriglyceridemia, low high-density lipoprotein cholesterol and abdominal obesity were 12, 15, 7, 8 and 9 respectively. Number of patients with degrees of stenosis between 30% and 69% suffered from hypertension, hyperglycemia, hypertriglyceridemia, low high-density lipoprotein cholesterol and abdominal obesity were 44, 34, 22, 30 and 35 respectively (Table 2).

**Table 2:** Metabolic Syndrome Distribution of Different Degrees of Intracranial and Extracranial Arteriosclerosis Stenosis

***Variable***	***Intracranial Arteriosclerosis n=105 (%)***		***Extracranial Arteriosclerosis n=87 (%)***	
	***Degrees of Stenosis***		***Degrees of Stenosis***	
	30%–69%	≥70%	30%–69% n= 68	≥70%
	n= 67	n= 38		n= 19
Metabolic Syndrome	30 (44.8)	26 (52.6) ▴	30 (44.1)	8 (42.1)
Components of Metabolic Syndrome				
Hypertension	46 (68.7)	34 (89.5) ▴	44 (63.8)	12 (63.2)
Hyperglycemia	38 (56.7)	30 (78.9) ▴	34 (50.0)	15 (78.9) ▴
Hypertriglyceridemia	26 (38.8)	19 (50.0)	22 (32.4)	7 (36.8)
Low High-density Lipoprotein	36 (53.7)	23 (60.5)	30 (44.1)	8 (42.1)
Abdominal Obesity	47 (70.1)	28 (73.7)	35 (51.5)	9 (47.4)
Metabolic Syndrome (Number of Component)				
1	10 (14.9)	4 (10.5)	15 (22.1)	4 (21.1)
2	16 (23.9)	8 (21.1)	9 (13.2)	5 (26.3)
3	13 (19.4)	15 (39.5) ▴	14 (20.6)	6 (31.6)
4	7 (10.4)	11 (28.9) ▴	8 (11.8)	5 (26.3)
5	3 (4.5)	7 (18.4) ▴	3 (4.4)	2 (10.5)

▴ *P* < 0.05, compared with degree of 30%–69%

### Correlation Analysis of Intracranial and Extracranial Arteriosclerosis and Metabolic Syndrome as well as Related Risk Factors

Intracranial Arteriosclerosis Group: After adjusting the factors including gender, smoking and coronary heart disease, a remarkable correlation between intracranial arteriosclerosis and metabolic syndrome (*P*<0.001) was detected. There was a correlation between intracranial arteriosclerosis and some components of metabolic syndrome such as hypertension, hyperglycemia and low high-density lipoprotein. Additionally, age and hyperhomocysteinemia were significantly related to intracranial arteriosclerosis (OR, 1.96 *P*= 0.01; OR, 1.89 *P*=0.007).

Extracranial Arteriosclerosis Group: After adjusting the factor of gender, no correlation between extracranial arteriosclerosis and metabolic syndrome was detected. There was a correlation between intracranial arteriosclerosis and hyperglycemia among the components of metabolic syndrome. Additionally, age, smoking habits and hyperhomocysteinemia were significantly related to intracranial arteriosclerosis as well. There was no correlation between extracranial arteriosclerosis and the number of components of metabolic syndrome (Table 3).

**Table 3:** Logistic Analysis of Intracranial and Extracranial Arteriosclerosis and Metabolic Syndrome as well as Related Risk Factors

***Variable***	***Intracranial Arteriosclerosis***			***Extracranial Arteriosclerosis***		

	***OR***	***95% IC***	**P**	***OR***	***95% IC***	**P**
Age(yr)	1.96	1.2–3.99	0.01	2.01	1.23–2.97	0.013
Gender	1.46	0.85–2.50	0.172	1.28	0.73–2.24	0.395
Smoking	1.13	0.94–2.12	0.056	1.68	1.24–3.00	0.005
Coronary Heart Disease	1.63	0.54–5.00	0.184	1.73	1.16–4.79	0.001
Hyperhomocysteinemia	1.89	1.23–2.56	0.007	1.93	1.34–3.65	0.006
Metabolic Syndrome	3.22	2.03–6.12	< 0.001	1.42	0.91–2.97	0.072
Components of Metabolic Syndrome						
Hypertension	2.05	1.78–5.13	0.012	1.57	0.94–3.23	0.065
Hyperglycemia	2.54	1.83–5.27	0.006	1.89	1.35–4.13	0.035
Hypertriglyceridemia	1.42	0.89–2.17	0.092	1.01	0.56–1.67	0.910
Low High-Density Lipoprotein	1.57	1.19–4.10	0.012	1.47	0.84–2.63	0.091
Abdominal Obesity	1.28	0.76–2.67	0.09	1.56	0.89–2.74	0.054
Metabolic Syndrome (Number of Component)						
1^b^	0.65	0.19–1.23	0.241	0.53	0.36–1.17	0.142
2^b^	0.77	0.42–1.46	0.511	0.41	0.26–1.06	0.151
3^b^	2.15	1.17–4.35	0.007	1.94	0.93–3.83	0.062
4^b^	2.27	1.03–4.54	0.022	1.83	0.84–4.21	0.091
5^b^	3.12	1.154–.45	0.023	1.79	0.54–5.45	0.288

a, control group as reference group;

b, 0 component of metabolic syndrome as reference group

## Discussion

Among 142 cases with recent cerebral infarction, 55% had a corresponding intracranial arteriosclerosis lesion (17). Extracranial arteriosclerosis was common for the western population, while the Asian, Blacks and Hispanic always suffered from intracranial arteriosclerosis (18–22). By continuously observing 318 patients with cerebral infarction and TIA, we discovered that the occurrence rate of intracranial and extracranial arteriosclerosis was 60.4%, among which intracranial arteriosclerosis accounted for 33.1% and extracranial arteriosclerosis accounted for 27.4%. Compared with existing results, the incidence rate of intracranial and extracranial arteriosclerosis was somewhat inconsistent. Though the lesion of intracranial arteriosclerosis is more than that of extracranial arteriosclerosis, the incidence rate of extracranial arteriosclerosis is on the rise, and the reason may be explained by the fact that in recent years we observed a significant improvement in life standard and the lifestyle (23).

We, showed that the comparison of gender between the intracranial and extracranial arteriosclerosis group and the control group had no statistical difference. The intracranial arteriosclerosis group and the extracranial arteriosclerosis group results were not in line with previous research results (24), because of the selected population. Patients enrolled in this study were over 60 years old (average ages were 67.9 years for male patients and 66.8 years for female patients). Estrogen in female patients could inhibit the arterio-sclerotic progress. A lack of estrogen due to surgery or other reasons is the most important element leading to arteriosclerosis for reproductive-aged females. Before 65 years old, the incidence rate of arteriosclerosis for females are obviously lower than males, however, after 65 years old, the incidence rate of arteriosclerosis for females and males are equal. Once stroke occurs in female patients, the results and prognosis are usually worse than those of the male patients belonging to the same age group (25). Here we showed that after losing the protection of estrogen, the risk of arteriosclerosis lesion for females is equivalent to males. Previous studies showed that smoking is an independent predictive factor for intracranial and extracranial arteriosclerosis (26), which can lead to arteriosclerosis by inducing vascular inflammation and oxidative stress (36). Meanwhile, vasomotor dysfunction, coagulation and fibrinolysis disorders and lipid deformation may appear, which can indirectly aggravate angiopathy (27).

Results of the present study showed that smoking did not have any significant correlation with intracranial arteriosclerosis, instead, there was a significant correlation between it and extracranial arteriosclerosis, which was consistent with those results obtained from previous studies (27, 28). For the patients with ischemic heart disease, it was very common to suffer from intracranial and extracranial arteriosclerosis, and a significant correlation was detected between the occurrence rate of extracranial arteriosclerosis and the severity of coronary arteriosclerosis (29). Coronary heart disease was not an independent risk factor of intracranial arteriosclerosis, instead, it was related to extracranial arteriosclerosis. The results were not only consistent with the results obtained in the studies conducted in Japan and Korean (30, 31). These results may explain the consistency of the correlation between extracranial arteriosclerosis and coronary heart disease exists among Asian population.

Among Asian and white patients, the relation between metabolic syndrome and intracranial arteriosclerosis is closer than extracranial arteriosclerosis (13, 14, 32, 33). We did not detect any correlation between different degrees of extracranial arteriosclerosis stenosis and metabolic syndrome. The reason why intracranial arteriosclerosis showed a significant correlation with metabolic syndrome might be as follows: 1. Under normal conditions, antioxidant enzyme contained in intracranial artery is superior to that of extracranial artery. Antioxidant enzyme in intracranial artery can decrease quickly with the age, which is more obvious compared with extracranial artery (34). It means that intracranial arteriosclerosis can speed up with age. This can explains the fact that age is an independent risk factor in intracranial arteriosclerosis in our study consistent with another study (35); 2. The main pathophysiology basics of metabolic syndrome is insulin resistance (IR) that can damage intracranial arteriectasia function (36) and lead to an increase in vascular wall stiffness and reduction of buffer ability. This increases the susceptibility of artery oxidative stress (37), and can speed up the formation of intracranial arteriosclerosis.

Hypertension is a clear risk factor that accelerates arteriosclerosis. The increase in systolic pressure and pulse pressure may increase the vascular shear stress and blood flow fluctuation. Therefore, the endotheliocyte can secrete NO, PGI, ET, leading to creation of an unbalance in angiokinesis material and exacerbation of endothelial cell damage. Compared with extracranial artery, the thickness and elasticity of tunica media in intracranial artery is the worse, so it can be more easily influenced by the vascular stress and blood flow caused by hypertension (38). Results obtained from other studies confirmed that the relationship between intracranial arteriosclerosis and hypertension is much closer, compared with extracranial arteriosclerosis (28, 39). The chief reason for diabetic attack is insulin resistance, and it has been verified that insulin resistance facilitate the incidence of arteriosclerosis. In the north of Manhattan and in Spain, diabetes was closely associated with intracranial arteriosclerosis (40, 41). Also, other studies (4, 24) on Asian population produced similar results. Hyperglycemia is an independent risk factor of extracranial arteriosclerosis, but no correlation between hypertension and extracranial arteriosclerosis was detected. This, suggested that pathoglycemia was a more important risk factor for extracranial arteriosclerosis compared with dysarteriotony.

High triglyceride and low high-density lipoprotein levels can promote the formation of low-density lipoprotein particles. Hypercholesteremia is a risk factor of intracranial arteriosclerosis (24, 42). In our study, high triglyceride levels did not show any correlation with intracranial arteriosclerosis, but low levels of high-density lipoprotein had an obvious correlation with intracranial arteriosclerosis. It has been shown that dyslipidemia has a close relation with intracranial arteriosclerosis, especially in Chinese population. There are also other studies showing that hypercholesteremia has a close relation with extracranial arteriosclerosis (43–45)

Visceral obesity is the basic constitution of abdominal obesity, which can result in an increase in free fatty acid release. An increase in plasma free fatty acid level plays an important role in occurrence mechanism of insulin resistance, induction of oxidative stress, and reduction of inflammation and blood vessel responsiveness. Moreover, visceral obesity can increase the secretion of very low-density lipoproteins, inhibit apolipoprotein B degradation and contribute to the formation of smaller low-density lipoproteins, which are closely related with the incidence of arteriosclerosis. Abdominal obesity showed a correlation with intracranial arteriosclerosis (14), however we did not find any correlation between abdominal obesity and intracranial and extracranial arteriosclerosis.

The results of Logistic regression in this study showed that the risk of intracranial arteriosclerosis occurrence would increase with an increase in the number of components of metabolic syndrome. This rising trend was not found in extra-cranial arteriosclerosis group, which is relatively consistent with the previous results (14, 33).

The drawback of this study lies in cross-sectional study, which only described the incidence rate of metabolic syndrome and the prevalence rate of intracranial and extracranial arteriosclerosis of patients who were admitted to our hospital from Sep. 2015 to Sep. 2017. The related risk between metabolic syndrome and intracranial and extra-cranial arteriosclerosis revealed in this study was very meaningful, providing a basis for target prevention and treatment on intracranial and extra-cranial arteriosclerosis lesion in the future.

## Conclusion

The high prevalence rate, death rate and disability confirmed the importance of the studies on intracranial and extracranial arteriosclerosis. This includes multicenter, large sample and prospective studies on intracranial and extracranial arteriosclerosis and risk factors, as well as the analysis of plaque image and pathology. Besides, intracranial arterial stenosis is an important reason for cerebral arterial thrombosis in China, therefore, seeking a reasonable treatment on intracranial arteriosclerosis has a far-reaching significance.

## Ethical considerations

Ethical issues (Including plagiarism, informed consent, misconduct, data fabrication and/or falsification, double publication and/or submission, redundancy, etc.) have been completely observed by the authors.
